# Shifting Social Norms and Adolescent Girls’ Access to Sexual and Reproductive Health Services and Information in a South African Township

**DOI:** 10.1177/10497323221089880

**Published:** 2022-05-20

**Authors:** Bronwen Gillespie, Julie Balen, Haddijatou Allen, Priya Soma-Pillay, Dilly Anumba

**Affiliations:** 1 Department of Oncology and Metabolism, University of Sheffield, Sheffield, UK; 2 School of Health and Related Research, University of Sheffield, Sheffield, UK; 3 Department of Obstetrics and Gynaecology, University of Pretoria and Steve Biko Academic Hospital, Pretoria, South Africa

**Keywords:** adolescent-friendly services, adolescent pregnancy, South Africa, cultural resistance, sexual and reproductive health

## Abstract

Despite policy on adolescent sexual and reproductive health (SRH) services and education, teen pregnancies remain common in South Africa. Social norms and cultural resistance are a well-documented challenge for SRH program implementation in South Africa, and beyond. To gain insight on the complex picture of adolescents’ access to SRH information and services in a peri-urban township, we explored this topic from a diversity of perspectives, carrying out 86 interviews to capture perceptions of adolescents, their parents, community members, and health professionals. Our research shows that despite the taboo nature of the issue of adolescent SRH, individual positions on adolescent access to SRH services and information are shifting and diverse, and are influenced by factors other than cultural norms. This research serves as a reminder to avoid simplistic reference to “culture” as a way of explaining health-related behaviors and people’s responses to health challenges.

## Introduction

Unsafe sex is the leading risk factor of female adolescent death worldwide (George et al., 2020). At least 10 million unintended pregnancies occur each year in women under 19 years of age in low- and middle-income countries (LMICs), as do an estimated 17,000 annual deaths from pregnancy or childbirth complications (Darroch et al., 2016). The 1994 International Conference on Population and Development helped put adolescent sexual and reproductive health (SRH) in the spotlight but implementation of adolescent-oriented services and programs remains uneven (Chandra-Mouli et al., 2019).

### Adolescent- and Youth-Friendly Services

Young people face specific barriers to accessing services. These include the widespread view that adolescents are healthy and not a priority category, or the tendency for youth to distrust and avoid services (WHO, 2002). This has led to a call for the development of “youth-friendly” services, more relevant and attractive to young people, with increased sensitivity to their needs (WHO, 2002). International guidelines such as *Making health services adolescent-friendly* (WHO, 2012) were published to standardize and scale up high-quality health services for adolescents, of which SRH care is a key area.

In South Africa, where this research takes place, efforts have been made to orient education and health policy to better respond to adolescent SRH needs. Girls aged 12 and over are eligible for free access to contraception without parental consent, and comprehensive sexuality education is part of the national education curriculum. The *Integrated School Health Policy* (Health and Basic Education, 2012) and the *National Policy on HIV, STIs and TB for Learners, Educators, School Support Staff and Officials in all Primary and Secondary Schools in the Basic Education Sector* (Department of Basic Education, 2017) mandate contraceptive services at school and sexuality education that covers topics such as gender, power, and relationships. It has also been recommended that adolescent- and youth-friendly services (AYFS) at community-level clinics should include after-school service hours for adolescents, and dedicated health staff trained in AYFS approaches, (Toska et al., 2019) but implementation remains a challenge. Adolescents have expressed dissatisfaction with SRH services, (Lake et al., 2019) and rates of unplanned pregnancies are reported to be highest among young, unmarried women (Kriel et al., 2019). These challenges are echoed in the international literature: in many cases, SRH interventions and programs do not reach the most vulnerable adolescents (Chandra-Mouli et al., 2015). A systematic review of indicators drawn from young people’s perspectives points to the importance of staff attitudes, including friendliness and respect, for adolescents across many contexts (Ambresin et al., 2013).

### Adolescent SRH and cultural resistance

In some settings, adolescent girls’ access to SRH services and information is hindered by adult views on adolescence and social norms^1^ surrounding youth sexuality (Chandra-Mouli et al., 2019; Starrs et al., 2018). Authors have found that cultural barriers such as taboos on sexuality and moral beliefs complicate communication on the topic across various LMIC contexts, including Zambia (Svanemyr, 2020), Tanzania (Kajula et al., 2014), India (Guilamo-Ramos et al., 2012), for Syrian refugees in Lebanon (El Ayoubi et al., 2021), and in Latin America (Campero et al., 2010; Nelson et al., 2014). “Culture” has been cited as a reason for limited adolescent SRH behavior change (Wight et al., 2012). Researchers have explored how social norms and the social environment influence youth SRH decision-making in the United States (Coley et al., 2013; Saftner, 2016) and numerous other settings in Africa (Challa et al., 2018; Pot, 2019; Svanemyr, 2020) and Asia (Shrestha & Wærdahl, 2020; Zuo et al., 2012).

Research on SRH across various African settings (Akwara & Idele, 2020; Sundewall & Poku, 2018), and further afield (Shariati et al., 2014; Thongmixay et al., 2019; Mollborn & Sennott, 2015), indicates the importance of managing cultural and social resistance to SRH programming. The cultural background of healthcare providers has been found to influence the way in which care is offered to adolescents (Yang, 2020; Pandey et al., 2019). The taboo nature of the topic can also make research a challenge (Jabareen & Zlotnick, 2021).

In South Africa, cultural taboos and people’s moral positions surrounding young women’s sexuality (Erasmus et al., 2020) have been shown to shape how the delivery of services unfolds in clinics (Jonas et al., 2019; Müller et al., 2016; Wood & Jewkes, 2006) and at schools (Francis & DePalma, 2014; Helleve et al., 2009). The moralistic and disciplinarian attitudes assumed by mothers (Mkhwanazi, 2014) limit engagement on this issue in the home. The literature indicates the importance of addressing community norms (Jonas et al., 2019) and recognizing the role of tradition, cultural beliefs, and religious faith (Ndinda et al., 2017). Given the growing policy awareness around AYFS programming, it is important to explore how adolescents and those involved in SRH service and information provision experience and manage enduring cultural barriers in this area, especially with adolescents in vulnerable socioeconomic settings, understood to be an underserved population (Santhya & Jejeebhoy, 2015).

We aim to explore the perspectives and everyday experiences of adolescents and community members (parents of adolescents, health services providers, teachers, and others) in a disadvantaged peri-urban township in Gauteng, South Africa, in regard to SRH services and communication, and the socio-cultural environment in which it is embedded. We refer to Fassin’s work on “culturalism” (2001) and Zigon’s (2009) discussion on morality to unpick the relationship between cultural resistance and adolescent SRH services and information at local level. Fassin (2001) used the example of maternity care in the Ecuadorian Andes to demonstrate that reference to culture should be a last resort in explaining limited uptake of health services and that culture-based theories can obscure socioeconomic or political factors, which may be the more crucial impediments to access to health. Zignon’s (2009) work on HIV in Russia explored how individuals interacted with existing social norms, and described a “moral breakdown,” when new circumstances arise which push people to reflect upon their everyday moral practice.

## Methods

### Study Design

Influenced by anthropological approaches, the study design focused on the social relations of those involved in seeking and offering SRH services and advice, as well as the context within which care takes place, to comprehend people and practices within broader social, political, and economic processes (Prentice, 2010).

The central research questions were how do adolescent mothers perceive of and react to existing services and information, and what are the reasons behind their use (or non-use) of available services and information? What are the factors, including the social environment, that shape how healthcare and information is offered, from the perspective of those involved? How can adolescent access to reproductive health information and services (specifically contraception) be improved?

The aim of the fieldwork was to collect enough data within a given setting to capture the complex picture of adolescents’ interactions with health services and other related sources of information, including the underlying factors that shape access to these services. This approach required the participation of different groups of people, in order to gain an understanding of the issue from a diversity of perspectives. These involved adolescent mothers and their parents (including parents of adolescents who were not mothers), young men, healthcare professionals and authorities, and community figures such as government officials, church leaders, and teachers. The study used ethnographic techniques. The bulk of the data was collected through semi-structured interviews, held in participants’ homes or work environments (clinics, schools, etc.). Specific semi-structured interview guides were designed for each group of participants (Table 1). We combined interview data with on-site observations of the context. While we did not have the resources for the long-term immersion typical of ethnography (Prentice, 2010), we carried out some participant observation in clinics and with community health workers on their neighborhood routes. This was included to gain a further sense of the context, taking into account that ethnographic techniques aim to consider human activity within the ordinary situations in which it takes place, to gain an “emic” view (Prentice, 2010).

**Table 1.** Summary of Topics in Interview Guides.

Type of Participant	Topics
Young mothers	Personal experience, narrative of before and after pregnancy and motherhood, and access to services and information
Young men	Views on adolescent pregnancy and access to SRH, perceptions of role of males
Parents (of adolescents)	Personal experience as a parent, opinions on adolescent access to SRH services and information
Healthcare professionals	Barriers to offering care, motivations, experiences, moral frameworks, challenges, and expectations
Community figures	Views on adolescent access to SRH services and information, own role and responsibilities, and perceptions of barriers
Health authorities	Program goals, perceptions of challenges and barriers

### Site Description

The study was carried out in a township on the outskirts of Pretoria bordered by informal settlements largely housing South Africans from other provinces and, to a lesser extent, people originating from neighboring countries. Townships are racially segregated neighborhoods designated for non-white residents under apartheid, usually on the periphery of towns or cities (Jürgens et al., 2013). Despite transformation since the demise of apartheid, this history has shaped the socioeconomic status of these areas. The site included more formal residential neighborhoods within the township, as well as the informal settlements between roads and up hillsides beside the township, largely composed of temporary dwellings with shared water taps, ad-hoc irregular electricity, and access through networks of unpaved pathways. Schools and clinics were attended by both residents of the township and the adjacent informal settlements.

The four health centers involved in this study offer family planning, as well as antenatal and post-natal services, while births and requests for abortion are referred to the district hospital. Condoms are available (male and female) without requiring staff consultation, and injectable contraceptives are the most commonly administered method. Adolescents may also encounter community health workers (CHW) in outreach teams who can screen (verbally) for pregnancy, direct mothers to post-natal clinics and offer counseling on contraceptive methods. Within some clinics, trained youth volunteers from a non-governmental organization also support these services.

### Data Collection

Data collection was carried out over a two-month period in mid-2019. The field team, which included an experienced international researcher (female), two research students in global health (one male and one female), and a multi-lingual research assistant with extensive local knowledge (female), carried out the interviews. Senior investigators based at the Universities of Pretoria and Sheffield supported the research design and analysis and writing phases.

With the assistance of outreach team leaders and community health workers affiliated with the four clinics, young mothers were identified and snowball sampling was used to find further participants (Table 2). Participants who had a first pregnancy before 19 years of age and those living in informal settlements were prioritized, given the study’s focus on socioeconomic disadvantage; however, some young mothers in formal residential areas were also interviewed to gain a range of perspectives. We sought out healthcare staff who were involved in offering SRH, and included community health workers whose designated visits included the informal settlements and households with young mothers. Most interviews were carried out in either English or Sotho, and detailed hand-written notes were taken during the interview. All notes in Sotho and other local languages were translated into English by the multi-lingual research assistant. To help assist full recall, details were added by the interviewer immediately afterward, and shared and discussed with the interview team, to ensure clarity. A full record of each interview was anonymized and entered into an electronic database. Citations presented in the results below are not guaranteed to be an exact verbatim record as audio recording was not used; however, every effort was made by researchers to accurately capture participants’ responses in their note-taking.

We chose to rely on note-taking, despite the potential loss of detail, to facilitate participants’ ease discussing sensitive matters. This was suggested by the local study team as more appropriate for the setting and nature of the research. The informal atmosphere enabled personal conversations of more depth. Young people and family members were interviewed in their homes or in outdoor patios. Interviewers shared snacks with participants and their family members, and used note-taking rather than recording equipment to create a more informal atmosphere. Female researchers carried out all interviews with female participants, and the male research student focused on the perspectives of the young men in the neighborhood.

### Description of Participants

We interviewed adolescent mothers served by four community-level clinics, parents of adolescents, other young women and men in the area, healthcare staff, health authorities (coordinators of government programs at city, municipal, and provincial level), and community figures (teachers, social workers, youth leaders, a local authority, and church leaders). Health care staff included clinic-based nurses and clinic directors (also nurses), outreach team leaders (nurses dedicated to supervising community health workers on home visits), and community health workers themselves (community members trained to offer preventative health advice, seek out patients who have missed treatment, and refer patients to the clinic after verbal screening, for example). All the healthcare professionals interviewed were female except for one outreach team leader. Table 2 lists the participants.

**Table 2.** Interviews Held.

Participant Group	Number
Young mothers (19 or under when pregnant)	30
Young women (who were not mothers)	5
Young men (who were not fathers)	10
Mothers of adolescent girls	10
Healthcare staff	12
Community health workers (CHWs)	5
Community members (5 male, 5 female)	10
Health authority staff	4
*Total interviews*	*86*

Thirty young mothers were interviewed, all of whom had become pregnant under 20 years of age, and all but two of whom were students at the time. At least half said that before their pregnancy, despite being sexually active, they had not visited the health center or used any form of contraception. A majority (25) of these births had taken place in the previous five years, and half of the babies were under a year old at the time of data collection. Eleven reported irregular or interrupted use of condoms or contraceptive injections previous to pregnancy. Almost all young women (90%) described their pregnancies as unplanned or unwanted. Most young mothers reported that after pregnancy, they were able to start using contraception, in part because they had become accustomed to visiting the health center, and several felt this was seen as more acceptable once they were already a mother, rather than as a sexually active adolescent.

Five young women who were not yet mothers, all students aged between 16 and 19 years of age, were also interviewed, as were 10 young men, ranging in from age 18 to 23 years of age, only one of whom was a father. Ten mothers of adolescents were also included, to gain the perspective of (grand)parents in the neighborhood. Fathers of adolescents were rarely available and less likely to be involved in everyday parenting. In the results section we label citations as collected from “young mothers,” who are those who became pregnant under 19 years of age, as opposed to “young women,” used when referring to adolescents without children, and to be distinguished from the generation of their own parents, labeled as “mother of adolescent” or “parent of adolescent.” We did not interview older mothers of young children as their experiences were outside the scope of this study.

Three focus group discussions (FGDs) with students attending two nearby schools were also carried out, in English and Sotho, lasting just over one hour each, as listed in Table 3.

### Ethical consideration

Ethical approval was granted by the University of Pretoria Faculty of Health Sciences Research Ethics Committee (137/2019) and The University of Sheffield Research Ethics Committee (UK). Permission to conduct the study was obtained through the Tshwane Research Office. Written informed consent was obtained from all participants prior to data collection. Participants aged 16 to 18 were involved only after receiving permission from themselves as well as their parents/guardians. Hand-written notes were anonymized, entered as typed files, and stored in an encrypted and password-protected computer.

### Data Analysis

Initial data analysis was conducted concurrently with data collection, with at least two researchers discussing each interview record to explore emerging themes. Data was triangulated through inquiring about the same topic with different participants, and by different researchers. In-depth inductive analysis was carried out after data collection was complete. NVivo (12 Pro) was used for data-driven coding based on emerging topics, for interview data from young mothers. The coding framework was created by listing topics after an initial review of the interviews, and modified after the first few interview files to include further significant topics. Analysis included a comparison of the topics across all files, and the topics in context of the specific interview, to take into account how the data related to the participant’s situation. For other participant groups where there were fewer interviews, coding was carried out within Word documents to understand the themes within the context of each interview and according to each type of participant. All participant groups were initially analyzed separately to observe differences and similarities within each group. We then noted the contrasts between narratives coming from the various participant groups. We paid special attention to how the different groups described their interactions with the other groups, and how their perceptions of challenges differed, given our interest in the social aspects around service provision (Table 3).

**Table 3.** Focus Group Discussions.

Focus Group	Number of Participants
Young mothers (all aged 18)	7
Young women (aged 16–18, not yet mothers)	4
Young men (aged 18)	6
*Total FGD participants*	*17*

Aspects of the interview guides focusing on the causes of adolescent pregnancy, the impact of motherhood, and the gender roles involved, pertaining to interviews with young men and women residing in the informal settlements, were reported in a separate but related paper (Gillespie et al., 2021) and therefore not repeated here.

## Results

The results are presented in sub-sections corresponding to the different participant groups. First of all, we present the factors as expressed by adolescents: Adolescents did experience challenges in access to services and information, linked in part to taboos on adolescent sexuality, including the challenge of communicating with parents and the fear of judgment from service providers. We then cover the views of parents of adolescents, who demonstrated a range of individual positions. While some refrained from offering SRH information as it was seen to promote sex among their adolescent children, others actively accompanied their adolescents to seek contraception. In the third section, we present the perceptions of a variety of community members, who do recognize the need for improved SRH for adolescents but feel that abstinence messages are preferred in the public sphere. Finally, the views of health service providers are set out, who observe that social taboos are only one of the limitations that shape how services are offered to adolescents. We found that social norms were being negotiated in some situations, and “culture,” though often named as a limitation to adolescent SRH services and information, was not necessarily the central obstacle.

### Adolescent Experiences of Taboos Limiting Access to SRH

Most adolescents said that discussions with their parents about dating and sexual relationships were of a disciplinary nature, warning them to focus on their studies. Several young mothers had hidden their sexual activity and pregnancy from family members. When asked about the appropriate age to let her family know it was time to go to the clinic for contraception, one teenager insisted: “No age! Never! You just don’t tell. If you are old enough you can go by yourself. They will wait until they see the baby arrive” (Young woman FGD1).

Several said that adolescents avoided these conversations, fearing scolding or shouting from parents. While a few adolescents reported having initiated conversations with their mothers, more often they had gained advice from an aunt or older sister. Many wished that the topic could be more easily addressed:

Mothers need to understand that teaching your child about prevention means you’re protecting them. It doesn’t mean you’re sending them to have sex, no. It just means you’re doing something so that they can continue their life and their education without anything stopping them. (Young mother M25B)

In this sense, adolescents did not express that it was wrong to be in a relationship or to use contraception; however, some feared scrutiny and judgment, which made them act secretively.

Several commented that they avoided visiting the clinic, imagining it could subject them to judgment from health services staff. This was in part due to the circulation of stories, as adolescents warn each other not to attend. Others had experienced verbal abuse directly, as seen in an account of a visit to the clinic, on suspecting pregnancy:

My first time at the clinic after I found out I was pregnant, someone shouted at me. The receptionist said, “You’re too young to be pregnant. Your boyfriend can’t even afford lipstick and you’re having babies!” It became so painful I started crying in front of everyone. (Young mother M5A)

Other adolescents managed to get regular contraceptive injections but described how services differed greatly depending on the clinic. “I don’t like Clinic C. Sometimes they shout. Sometimes you have to go home, they say return another day. So, I went to Clinic D with a friend. They were nice” (Young mother M30C).

At school, social norms were also seen to limit how SRH education took place. Most adolescents, male and female, did report receiving some useful SRH information within Life Orientation classes. Many young women, however, felt it lacked practical detail. One adolescent felt her teacher appeared uncomfortable talking about certain topics, while another said the focus in class had been on abstinence. Young women desired further reliable information about the various methods available. It was common to share complaints about methods and about health services between friends, but this was not necessarily discussed in the classroom—adolescents did not have accurate knowledge of how contraception actually worked. Several young women said they stayed away from contraception fearing weight gain, while both young men and women had heard it caused infertility.

### Parents of Adolescents: Shifting Positions on Adolescent SRH

In the first instance, parents of adolescents placed high value on abstinence and most were not open to discussing sex and contraception with their adolescent children. Mothers actively warned their daughters away from “dating,” understood locally to quickly lead to sexual activity, and many repeated the sentiment that 21 is the age to start dating, and 30 to start a family. Parents of adolescents said they were aware that secret dating was widespread but that they found it difficult to discuss outright, as summed up in one mother’s description of the dilemma: “My 15-year-old, I suspect she has a boyfriend but I don’t know if she is sexually active. My challenge is if I start sending her there [to the clinic], I will allow her to have sexual activity” (Mother of adolescent P2).

Many parents of adolescents expressed concern about how best to ensure their own children avoid unwanted pregnancy. Some claimed to be actively confronting the taboos around acknowledging adolescent sexual activity. One of the mothers, noting the “unaccounted disappearing” of her 14-year-old, acted beyond the norm and took her to a doctor to initiate contraception. For some, desire for their child’s future success motivated action on contraception. One mother spoke of her plans for her 17-year-old daughter: “I started my girl on contraceptives. I hope she will take her studies seriously and become a professional and be independent” (Mother of adolescent P10).

Others spoke about how they felt unable to take such decisive action. One mother explained, “I talk to them about condoms, it is so embarrassing at times to talk about this. I wish as parents we could be bold and take our girl to the clinic the minute we suspect she is dating” (Mother of adolescent P9). Several mothers of adolescents, now grandmothers, expressed regret and disappointment of having failed to ensure their daughters’ contraception use to avoid early pregnancy. Recognizing parent–adolescent communication barriers, several expressed that SRH education should be the domain of the school:

They should give more education . . . maybe approach it differently. The more education they give to the kids, the better. They are more comfortable with teachers. Maybe they should invite outsiders to come teach about family planning, health issues . . . The teaching should be explicit. (Mother of adolescent P2)

Overall, we found that moral beliefs around adolescent sexual activity did not appear to impede adults from valuing adolescent access to SRH services. However, there was a wide range in their capacity to offer information or support service use, from those who did not know how to talk about it, to others who even managed to accompany their daughters to the clinic.

### Community Members’ Views on SRH Information

Community members, including government officials, church leaders, and teachers, felt that messages emphasizing abstinence were generally preferred by members of the community, including parents of adolescents. This was seen to limit what could be said in public, undermining SRH-related health campaigns. One local authority explained:

Our cultures, pushed through the church, have a heritage that says abstinence . . . The government can’t overlook this. We need to be on the side of the people, and the people are with the church . . . The government comes with a law and says “push this” so we do it without believing, because we know it won’t settle in the community. (Local authority CM4)

Church authorities themselves reported that they were compelled to insist on abstinence for youth when speaking in public, but commented that they had come to realize that young people should also be able to gain advice on contraception, for example, through church youth camps or volunteer counselors, and were not averse to the topic being covered in private.

Teachers felt that social norms did sometimes dictate what could be offered and they tailored classes accordingly. “There is a limit. There was an organization that wanted us to distribute condoms and I said no. It’s like we are promoting sex. As a parent I did not want to do that on the school grounds” (Female teacher CM7). They were aware that sexuality education was not being covered in sufficient detail, but more than their own moral position, this was out of a sense of what was acceptable for students’ parents. One teacher observed that she felt limited by parents’ opinions saying, “It becomes a moral issue, for the community . . . before you are a teacher, you are a parent” (Female teacher CM6). They felt that efforts to expand curriculum implementation would require a concerted effort to coordinate between the Department of Basic Education, teachers and school governing bodies to get students’ parents on board.

Teachers also felt that it was difficult to cover all relevant material in depth, due to lack of time and resources. They suggested that more support was required to improve SRH programs, and recommended more input from school nurses. It was noted that clinic-based school nurses very rarely have time to work on SRH. In one teacher’s words:

The Board of Education notices that pregnancy statistics are getting higher. They say, “You must support the girls so that they can continue school.”^2^ But when will I do that? And when will I focus on prevention? The school just tells the girls to go to the clinic . . . I’m everything – teacher, security, social worker, I do everything! (Female teacher CM7).

### Healthcare Staff Working within Limitations

In regard to the health care sector, we found a wide awareness of the need to ensure non-judgmental AYFS. In the words of one health program manager:

We have an issue with “attitude”. Learners are advised to go to nearest clinic . . . But the learner arrives and comes across this “mother” attitude. It’s part of the culture . . . Some nurses get upset, shout, want to punish because they don’t condone what teenagers are doing. (Health program manager A4)

However, from those involved directly in offering care, institutional or system-wide issues, rather than cultural beliefs or moral positions, were undermining the implementation of AYFS. The nurses described their commitment to increasing adolescent access to contraception, and many felt that attitudes within the profession had changed, and that staff were prepared to support adolescents. As one nurse commented: “I have to separate my morals from my job . . .” (Female Nurse N5A). Through AYFS training, they had come to understand the importance of offering non-judgmental services:

I have to be comfortable with them. Especially the teenagers. If you become polite and friendly, then they will open up. If you sit and say, “I’m the Sister” [title for professional nurses] then they will do no such thing because you’ll be like a parent to them and they’re already scared of their parents . . . It’s very important to have that open relationship . . . Now they request me at the reception. It’s easier for them. (Female Nurse N7B)

Several healthcare providers noted concerns that receptionists did not receive the same training, describing this as a significant barrier for adolescents, since the receptionists are usually the first point of contact.

Several critiqued the policy that enabled adolescent students a “fast-track” instead of waiting in line, as it caused tensions with other patients who had been waiting all day for services. A service model (implemented in some provincially run clinics) with a specific adolescent-dedicated service (for all health concerns) was preferred, as explained by one nurse in a managerial role:

We need a teen section. If I had more resources, I would make a special section for teens, if they are sick, or for family planning, or HIV. They are embarrassed to be in the line for HIV. Teens could come get family planning without being in the line with mothers. They don’t want to go to “gogos” [grandmothers]. (Female Nurse N14B)

The adolescent-specific service, as opposed to the “fast-track” (within general maternal and SRH services) was seen to offer improved privacy as adolescents would not be waiting with other community members, nor would the motivation for their visit be revealed, not even to the receptionist. Furthermore, younger staff are more often chosen to be trained in AYFS, so that adolescents are more comfortable. One nurse compared her working environment to a provincially run clinic:

The clinic in W is so well-run and well-staffed. They have a professional nurse who only deals with AYFS . . . She’s not doing ANC [antenatal care], curative, or squeezing people in . . . AYFS works better at the province because you have somebody who’s responsible for youth. With us, we have to stretch ourselves and cover all other services. (Female nurse N6A)

Many nurses felt that understaffed clinics limited the availability of viable services for adolescents, and drew attention to the general overburdened working conditions of nurses, felt to have deteriorated and caused lack of motivation in staff. As observed by teachers, health authorities felt that combined health and education sector policy implementation was lagging due to limited resources and required improved commitment to cross-sector collaboration. One nurse commented: “I believe we need the three parties on board: teachers, parents and health [care staff]” (Female Nurse N2D).

Overall, we found an impasse in communication around SRH issues. Though grounded in the cultural backdrop of taboos around sexuality, this does not tell the whole story. Parents of students are said to block sexuality education, yet they call for more attention to the topic in schools. Adolescents feel the topic is taboo, yet parents say they are trying to talk about it. Healthcare staff are committed to meeting contraceptive needs, yet many adolescents still face limited access to services. Issues around resources were raised, as was the importance of coordination and commitment from health and education sectors.

## Discussion

In this study, we explored adolescent perceptions and use of SRH services and information, and found that many described limitations and strains, including challenges in talking to their parents and avoidance of services, due to fear of judgment for being sexually active as adolescents. We examined factors involved in how services and information are offered, and found that parents face uncertainty in communicating about SRH issues, weighing between a tradition of abstinence talk, and emerging positions that favor direct advice and action. In contrast, community members emphasized parents’ lack of support for SRH information provision as a main limitation. In the clinic setting, institutional factors such as understaffing were seen as more problematic than cultural attitudes, which had shifted. To improve adolescent access to SRH, service providers in health and education sectors called for prioritization of this area, as their interest in responding to adolescents’ needs was undermined by lack of time and support. Improvement was also seen to rely on increased communication between the various interest groups involved, to publicly open up debate and move beyond lingering taboos.

Perceptions on adolescent SRH information and services were found to be diverse and evolving, and factors above and beyond cultural taboos were at play in limiting access to SRH support. We therefore argue that simplistic references to cultural or social norms as a means of explaining health-related behavior can be imprecise, and may hinder a more nuanced inquiry. First of all, we concur with Fassin’s (2001) position on “culturalism” and the danger of assuming culture as explanatory. Secondly, we observe that culture is dynamic: with reference to Zignon’s (2009) work on morality, we see how cultural resistance surrounding the topic of adolescent sexuality is being re-thought on a personal or internal level, if not yet in public. Thirdly, we note that a focus on culture must take care not to avert important attention away from the recognition of diversity of positions. Finally, we propose that a nuanced approach to research on socio-cultural barriers can serve to detect opportunities to help promote improvements.

### Using Culture as Explanatory can Obscure Other Factors

Community members, healthcare professionals, and health authorities did indeed cite tradition and refer to “our culture” and the disciplinary “mother” attitude found in clinics, when explaining why adolescents could not always easily visit health clinics. Similarly, existing literature also documents these cultural barriers (Erasmus et al., 2020; Jonas et al., 2019; Müller et al., 2016). However, we also found that those closely involved in providing services or information to adolescents went on to emphasize other non-cultural challenges. Teachers and nurses involved cited that lack of support and limited resources undermined efforts to offer adolescent SRH services and education. Nurses were clear about their commitment to ensuring adolescent access to contraception, and described how training programs had led to changes in attitude among staff. Our research therefore reflects Fassin’s (2001) warning that culture-based theories can obscure socioeconomic or political factors, which may be the more crucial impediments in access to health, and that reference to culture should be a last resort in explaining limited uptake of health services. For example, research in the United States critiques healthcare providers’ assumptions that Latinas delayed SRH care-seeking is “cultural,” noting that overlooked barriers included the expense, time commitment, language divide, and negative stereotypes encountered, as well as other system-level factors (Guerra-Reyes et al., 2021). Fassin’s position has also been used in research in Malawi, which observes that “culture” is overemphasized as an explanation for high rates of teen pregnancy, when this is tied closely to socioeconomic and political circumstances (Pot, 2019). For our work in South Africa, this perspective is relevant for helping to unpick assumptions that the cultural environment is responsible for the lack of improvement of adolescent access to SRH care and services.

A focus on cultural beliefs may inadvertently appear to blame nurses and other service providers for not shifting their attitudes towards adolescents, taking attention away from structural factors that limit their ability to effectively deliver services. Though we do not deny that attitudes can pose a challenge, both healthcare professionals and teachers themselves offered perspectives which imply that problems in access to services and information are not simply an enduring cultural issue. Along these lines, studies in South Africa point to the institutional challenges facing healthcare professionals, including staffing shortages and a lack of prioritization of SRH (Jonas et al., 2019; Pillay et al., 2020). AYFS has been found to lack the required support for facilities to attain desired standards (James et al., 2018).

A focus on culture is also problematic without due attention to the conditions under which culture is produced and reproduced (Fassin, 2001). For example, building on Fassin, Hlabangane (2014) points to the danger of overstating the links between black “culture,” tradition, and notions of sexuality as drivers of HIV in Africa, as this serves to reify cultural difference without necessary consideration of the role of enduring structural exclusion (Hlabangane, 2014). In this sense, although nurses’ moral positions on adolescent sexuality are an important obstacle (Müller et al., 2016; Wood & Jewkes, 2006), understanding the complex roots of nurses’ disciplinary behavior is important. Researchers have highlighted how verbal abuse and judgmental actions in current-day healthcare provision in South Africa (Zerucelli Rucell, 2017) can be traced to the historical organization of healthcare provision and the legacy of apartheid that located black nurses at the bottom of the scale, so that power inequity shaped the care they offered. Furthermore, the historical opportunity for non-whites represented by the nursing profession (Varga & Brookes, 2008) positioned nurses in a specific social role, as guardians of morality in the community, feeding into the disciplinary dynamics seen in care today.

### Using Culture as Explanatory Disregards its Dynamic Nature

Although many adults expressed uncertainty and wariness about communication with adolescents on SRH, this position was not static, nor were social norms necessarily deterministic of actual individual behavior. With reference to Zignon’s (2009) work on morality and culture, we look at how individuals have diverged from social norms as they respond to the urgent need to respond to adolescents’ everyday reality.

For Zignon, morality is not congruent with culture. Individual moral practice can differ from morality at the discursive level—public and institutionally articulated discourses, such as those of an organized religion, may influence, but not determine, individual practice (Zigon, 2009). Writing on HIV in Russia, Zigon (2009) describes what he calls a “moral breakdown,” or the “stepping away” from usual everyday moral practice, prodded by new circumstances or events which problematize habitual moral positions (Zigon, 2009). This situation echoes Parish’s observations that morality arises “not from conformity to discourses or codes that are labelled ‘moral’, but from a painful questioning of self and culture that potentially revises acts of moral valuing” (Parish, 2014:34). Parish (2014) sees morality as grounded in experience. People confront their own position as new events (for example, adolescent dating and sex) intrude. In this South African Township, discursive morality on teen sex was clear: “dating at 21 and marriage at 30” was heard in many households. Yet, some mothers were surreptitiously urging their much younger daughters to visit the clinic, or even bringing them there.

This parenting behavior speaks to women’s recourse to pragmatism in health-related decision-making (Lock & Kaufert, 1998). Lock and Kaufert’s work on “reproductive pragmatism” has contributed to understandings of women’s agency and behavior within shifting choices in the context of globalization, drawing attention to how women evaluate reproductive options in light of their personal, social, and economic circumstances (Wild et al., 2015). As the authors explain, women will experiment and adopt new measures, if the apparent benefit outweighs the cost (Lock & Kaufert, 1998). In our study, some mothers felt it was worth defying the stigma on adolescent sexuality, while others bemoaned that they had not pushed past their discomfort and spoken to daughters more openly. Previous ideas of being a good mother—not talking about sex and disciplining daughters who were sexually active (Mkhwanazi, 2014)—are giving way as adults realize that new measures of protecting their adolescent girls from unwanted pregnancy are required. Similar to our research, work in SRH in India critically assessed widespread assumptions that due to their cultural context, families would be unwilling to discuss sex-related topics. It was found that parents and adolescents wished for more open conversation on SRH, especially in light of worry surrounding pregnancy, HIV, and sexually transmitted infections, but felt limited by a lack of information and embarrassment (Guilamo-Ramos et al., 2012). We can see that parents’ health concerns for their adolescents outweigh pervading norms to shape a new pragmatic stance.

Regarding health professionals, compared to some earlier studies (Müller et al., 2016; Wood & Jewkes, 2006), we found nurses prepared to promote adolescent access to contraception and clear that this was within their professional responsibility. For some, the reflective process was linked to professional training. For others, nurses (and teachers), the desire for improved access to contraception was fed by their everyday experiences with adolescents facing unplanned pregnancy. Coping with the reality of adolescent pregnancy in their professional settings was enough to push their “morals aside”—morals that had become unsuitable for new circumstances (Zigon, 2009).

### Using Culture as Explanatory Undermines the Diversity of Positions

We found that although taboos were present, they were not necessarily actively upheld by all, raising important questions about exactly whose positions are represented when referring to “culture.” Community members commented that parents generally preferred messages that promote abstinence, as part of cultural and religious affiliation, whereas interviews with parents showed that this general impression of parents’ positions does not accurately represent everyone’s interests. This serves as a reminder of the importance of paying attention to a multiplicity of voices (Grillo & Stirrat, 2020), to take note that some voices are more likely to be heard and to carry more weight. Explanatory models referring to local cultural beliefs must be cognizant of which people are speaking the loudest, influenced by whom, and for what reasons.

Our research also points to the importance of recognizing the multiple or overlapping positions of those involved. Crenshaw’s (1990) work on intersectionality—the interaction of different axes of identity (such as gender, ethnicity, and age) dependent upon context and setting—has been used to emphasize the importance of health systems solutions that take individuals’ multiple social locations into account (Hankivsky et al., 2014). Positions were seen to vary according to the role people assumed, as parent or professional, in public or private. Nurses and teachers both raised observations about the tensions in their different identities, and questions regarding upholding their moral positions.

Finally, culture may also be used by those involved as a discursive resource (Macleod et al., 2011) to serve particular interests. This has been documented in South Africa to use cultural arguments to oppose abortion, for example (Macleod et al., 2011), for strategic and political ends. In our research, “our culture” was brought up by those in positions of authority to explain why services are offered in a certain way, or why adolescents tend to expect disciplinary treatment. Though this can to some extent reflect reality, it can also be seen as an umbrella term that serves to sidestep more rigorous critique of why certain institutional problems endure.

### Detecting Opportunities

We do not intend to discourage investigation on cultural norms, but rather to promote it as an entrance point to gain further insight. Research in this area can shed light on the way that cultural assumptions may inadvertently draw attention away from more complex structural aspects. Health professionals described changes in attitudes, and testified to the benefit of training on adolescent-friendly services, but were frustrated with limitations encountered in everyday clinic operation. This research has clarified the essential requirement of investing in nurses and teachers who are directly in contact with adolescents and comprehend the everyday realities of adolescent pregnancy. Service providers require committed leadership from health and education authorities to help them negotiate enduring taboos and act to prioritize adolescents’ needs.

The way norms are viewed or managed by different actors yields a rich area of study. Looking at the diversity of positions on social taboos can help to detect hidden opportunities such as shared interests from which to open dialogue on sensitive topics. For example, even though parental resistance is a valid challenge, it does not accurately explain the views of all parents of adolescents, and cannot be used as a scapegoat for limited SRH education in schools. Understanding a variety of actors’ opinions on taboos helps to cut across assumptions, identify allies, and facilitate collaboration. Reducing the gap between adolescent-oriented government policy on paper and the services that are actually available and taken up at ground level requires in part a voicing of demand for these services, which is complicated for stigmatized topics. Uncovering opinions which are not yet part of the public domain can help to foster critical support for tackling issues related to vulnerable or less-heard groups, such as sexually active adolescent girls.

### Limitations

All of the findings of this study may not be generalizable to all settings, as it was carried out in a specific neighborhood where health service challenges were partially shaped by overlapping management jurisdictions, the full impact of which was beyond the scope of this research. As well, the study did not interview adolescents under the age of 16, nor those under the age of 18 whose parents were not available to give consent.

## Conclusion

We found that both in the literature and in the study setting, much is made of cultural grounds for rejection of adolescent access to SRH information. However, examining how local people engage with cultural barriers shows that positions are nuanced and fluid. We suggest that pragmatic decision-making can shift the boundaries of people’s moral stance, making us wary of emphasizing moral or cultural opposition to SRH goals. Research that explores these dynamics can serve to detect opportunities to further support tentative transitions already underway. Helping adolescents avoid early unwanted pregnancy has increasingly become a pragmatic imperative at community level, and we suggest that committed health and education sector leadership and investment would facilitate progress and help overcome enduring resistance. This research serves as a reminder to avoid using “culture” to explain health-related behavior as it may obscure other causes and fail to accurately represent the shifting nature of culture nor the diversity of views that shape people’s reactions to health challenges.

## Notes

1. Social norms are the largely unwritten or informal rules that differ from culture to culture, and make up what’s seen as normal, acceptable behavior.

2. For learners who become mothers, policy facilitates continued school attendance [Department of Basic Education, 2017]. Teachers reported improvements in retaining young mothers.
